# A Case of Significant Response to Olaparib in a Patient with Primary Peritoneal Carcinosarcoma Diagnosed by Laparoscopic Surgery

**DOI:** 10.1155/2020/9106390

**Published:** 2020-08-10

**Authors:** Satoshi Nagamata, Yukiko Nakasuji, Keitaro Yamanaka, Maho Azumi, Keiichi Washio, Maho Shimizu, Ryosuke Takahashi, Kazuyo Matsumoto, Yuka Murata, Kaho Suzuki, Masashi Deguchi, Hideto Yamada, Yoshito Terai

**Affiliations:** ^1^Department of Obstetrics and Gynecology, Kobe University Graduate School of Medicine, 7-5-1 Kusunoki-cho, Chuo-ku, Kobe, Hyogo 650-0017, Japan; ^2^Department of Obstetrics and Gynecology, National Hospital Organization Kobe Medical Center, 3-1-1 Nishiochiai, Suma-ku, Kobe, Hyogo 654-0155, Japan

## Abstract

Primary peritoneal carcinosarcomas which arise from extragenital locations are extremely rare. Carinosarcomas contain both carcinomatous and sarcomatous elements and can be mainly detected in the female genital tract. We herein report a case of primary peritoneal carcinosarcoma diagnosed by laparoscopic surgery and treated with olaparib. A 62-year-old woman referred to our hospital due to abdominal distension. From imaging findings, we suspected advanced primary peritoneal carcinoma, and laparoscopic surgery was thereafter performed. The pathological diagnosis was carcinosarcoma, and the patient received chemotherapy with docetaxel and carboplatin. After three cycles of chemotherapy, the interval debulking surgery was attempted but resulted in suboptimal results. Because the bilateral ovaries were observed with a normal size and normal findings, we considered that the most likely diagnosis was primary peritoneal carcinosarcoma. After the additional chemotherapy and a 6-month observation period, the tumor relapsed. The patient received chemotherapy again, and the peritoneal carcinosarcoma was judged to be a platinum-sensitive tumor. Oral administration of olaparib was thus initiated. Although a dose reduction was needed due to anemia, olaparib was effective, and the patient could continue the drug for another 7 months. This is the first report of primary peritoneal carcinosarcoma treated with olaparib and shows that it could be a treatment option for platinum-sensitive tumors.

## 1. Introduction

Carcinosarcoma is a malignant biphasic neoplasm composed of carcinomatous and sarcomatous elements. It is also called malignant mixed Mullerian tumor (MMMT) and usually arises from the female genital tract, including the ovaries, uterus, and fallopian tubes [1].

Primary peritoneal carcinosarcomas which arise from extragenital locations are extremely rare [2], and a small number of cases have been reported where carcinosarcomas occur in the Douglas' pouch [3], retroperitoneum [4], mesentery [5], and lesser omentum [6]. Primary peritoneal cancer and ovarian cancer share a common embryological origin. The inclusion of primary peritoneal cancer within the ovarian epithelial cancer designation is generally accepted, and the therapeutic strategy for primary peritoneal cancer follows the advanced ovarian cancer regimen [7]. Olaparib is the first poly ADP-ribose polymerase (PARP) inhibitor to be approved in Japan and used for maintenance therapy for platinum-sensitive relapsed ovarian cancer, fallopian tube cancer, and primary peritoneal cancer [8]. However, olaparib therapy for primary peritoneal carcinosarcoma has not yet been reported.

Minimum invasive surgery by laparoscopy provides direct visualization and adequate tissue sampling for the histopathological diagnosis of cancers in the abdominal cavity [9]. We herein report a case of primary peritoneal carcinosarcoma diagnosed by laparoscopic surgery and treated with olaparib. It is the first such report of its kind.

## 2. Case Report

A 62-year-old woman, gravida 4, para 2, presented with abdominal distension. Her past medical history was hypertension and hyperlipidemia. She also had a family history of lung cancer in her father, yet no family history of breast or ovarian cancer.

**Figure 1:**
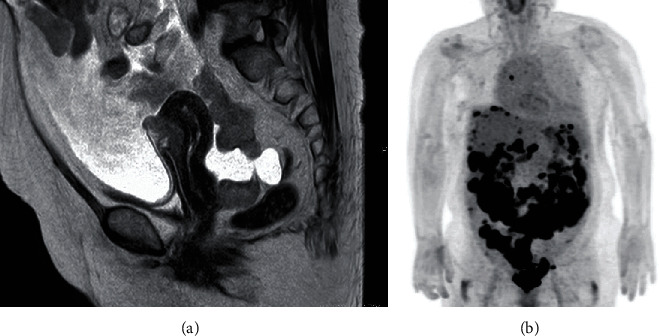
(a, b) T2-weighted magnetic resonance imaging of the pelvis and 18F-fluoro-2-deoxy-d-glucose positron emission tomography/computed tomography revealed massive peritoneal dissemination, including in the Douglas' pouch, vesicouterine pouch, omentum, and the surface of the liver and spleen. Bilateral ovaries were not clearly enlarged and were not visualized in the image.

Transvaginal sonography revealed massive ascites and tumor dissemination in the Douglas' pouch. Laboratory data revealed that CA125 at 789 U/ml, LDH at 562 U/l, and other tumor markers (CEA, CA19-9, and SCC) were within normal limits. Magnetic resonance imaging (MRI) and 18F-fluoro-2-deoxy-d-glucose positron emission tomography/computed tomography (FDG PET/CT) revealed massive peritoneal dissemination, including in the Douglas' pouch, vesicouterine pouch, omentum, and the surface of the liver and spleen (Figure 1). Bilateral ovaries were not clearly enlarged and were not visualized in the image. Pleural effusion and parasternal lymph node metastases were also found.

**Figure 2:**
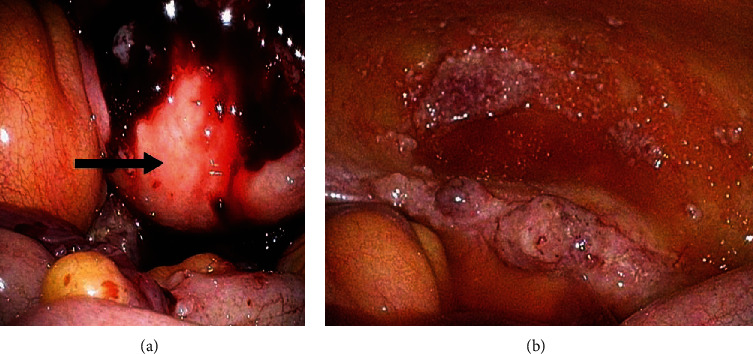
(a) Laparoscopic surgery was performed for the biopsy. The bilateral ovaries were not seen due to the frozen pelvis. The arrow indicates the uterus. (b) Laparoscopic findings of the peritoneal dissemination in the vesicouterine pouch.

From these imaging findings, we suspected advanced primary peritoneal carcinoma. In order to make a diagnosis of the tumor, laparoscopic surgery was performed. Laparoscopic findings documented the dissemination in the whole of the abdominal cavity, but the bilateral ovaries were not seen due to the frozen pelvis (Figures 2(a) and 2(b)). A laparoscopic biopsy was then taken from the tumor of the peritoneal dissemination.

**Figure 3:**
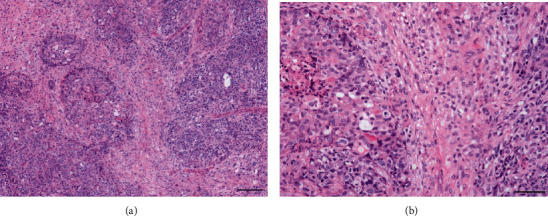
Examination of hematoxylin and eosin-stained sections revealed that the tumor was composed of malignant epithelial elements and sarcomatous elements of spindle cells. (a) The epithelial component displayed the solid proliferation of severely atypical cells, thus mimicking ovarian high-grade serous carcinoma. (b) The stromal component consisted of atypical spindle cells mimicking leiomyosarcoma. Scale bars: (a) 200 *μ*m and (b) 100 *μ*m.

**Figure 4:**
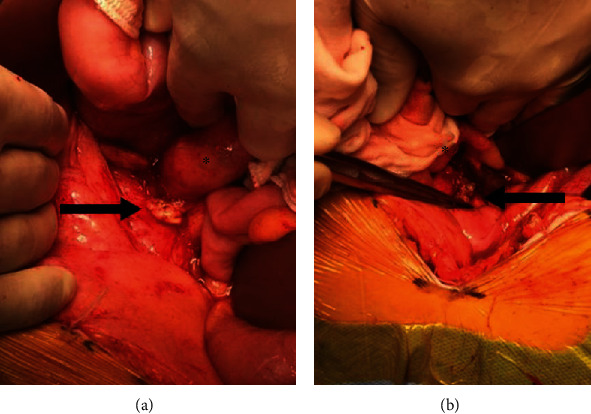
(a, b) The bilateral ovaries could be observed within a normal size and normal findings during the laparotomy after three cycles of chemotherapy. The arrows indicate the ovaries, and the asterisk indicates the uterus. Therefore, the diagnosis was confirmed as primary peritoneal carcinosarcoma.

Histological examination showed that the tumor was composed of malignant epithelial elements and sarcomatous elements of spindle cells (Figure 3). The epithelial component displayed solid proliferation of severely atypical cells, thus mimicking ovarian high-grade serous carcinoma, and the mesenchymal element resembled leiomyosarcoma. Immunostaining revealed positive staining for paired-box gene 8 (PAX8), for Wilms' tumor-1 (WT-1), focally for Ber-EP4, and for MOC31 in only the serous carcinoma components. It was diffusely positive only for p53. The pathological diagnosis was carcinosarcoma of a homologous type, and the patient received chemotherapy with docetaxel and carboplatin due to her acute hypersensitivity reaction to paclitaxel. The patient received carboplatin at a target area under the curve (AUC) of 5, according to Calvert formula, together with 75 mg/m^2^ docetaxel every 3 weeks. Due to grade 4 neutropenia and grade 2-3 nausea, fatigue, and myalgia, the doses of carboplatin and docetaxel were reduced to AUC 4 and 60 mg/m^2^, respectively, after 2 cycles. After three cycles of chemotherapy, CT revealed a partial response with a 47% decrease in the sum of the longest diameter of target lesions according to the Response Evaluation Criteria in Solid Tumors (RECIST) [10]. Although interval debulking surgery was attempted, tumor dissemination remained in the abdominal cavity, and we therefore performed an omentectomy only. Regardless of the strong adhesion in the Douglas' pouch, the bilateral ovaries could be observed to be within a normal size and with normal findings (Figure 4). We therefore considered that the most likely diagnosis was primary peritoneal carcinosarcoma.

**Figure 5:**
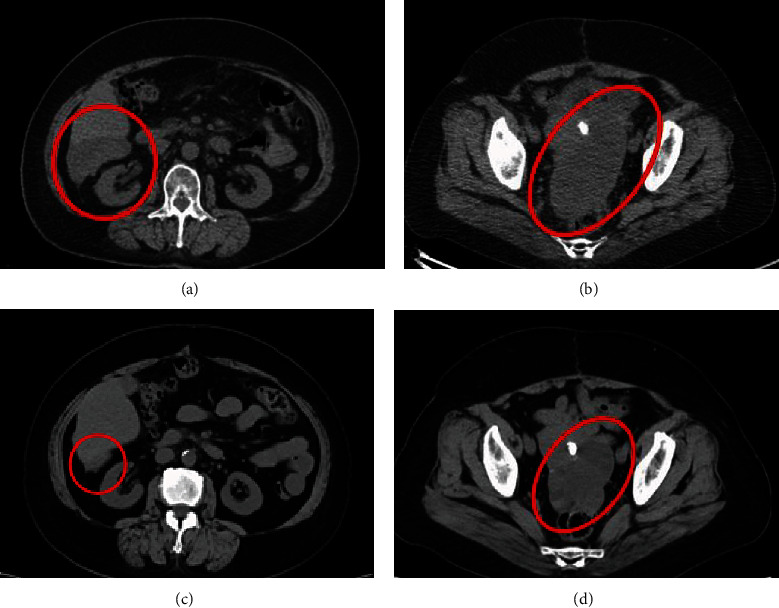
(a, b) Computed tomography revealed peritoneal dissemination of the surface of the liver and Douglas' pouch. (c, d) After four months of olaparib administration, computed tomography revealed a decrease in the size of the tumors.

After the surgery, CA125 dropped to 28 U/ml, and the patient received three cycles of docetaxel and carboplatin again. A subsequent FDG PET/CT scan showed that the FDG accumulation in the whole body had completely disappeared. A tumor marker follow-up revealed that CA125 had lowered to 10 U/ml. We then recommended a secondary debulking surgery with intestinal resection, but the patient did not desire to have the surgery. After a six-month observation, peritoneal dissemination relapsed. The patient then received three cycles of docetaxel and carboplatin, and a partial response with a 32% decrease in the sum of the longest diameter of target lesions was confirmed by CT. Her serum CA125 level dropped from 468 U/ml at recurrence to 29 U/ml. The peritoneal carcinosarcomas were judged to be platinum-sensitive tumors. However, the patient suffered from the adverse effects of chemotherapy, such as grade 2-3 nausea, fatigue, and myalgia, and therefore, the oral administration of olaparib tablets 300 mg twice daily (total 600 mg everyday) was initiated based on the patient's sensitivity to platinum and only after fully informing her and her family about the off-label use of the drug. Due to her anemia, the dose of olaparib was reduced gradually to 200 mg twice daily (total 400 mg everyday). However, after four months of olaparib administration, CT revealed a significant response with a 43% decrease in the sum of the longest diameter of target lesions compared with the CT before the olaparib administration (Figure 5). The serum CA125 level had decreased to 13 U/ml after 2 months of olaparib administration. Furthermore, no side effects were observed after reducing the dose of olaparib, and in total, the patient could continue olaparib treatment for 7 months.

After 7 months of olaparib administration, CA125 had gradually increased to 65 U/ml, and an FDG PET/CT scan revealed a relapse of peritoneal dissemination. The patient received three cycles of docetaxel and carboplatin again. However, CT revealed the progression of the disease, and massive ascites had reappeared. Although the patient received one cycle of pegylated liposomal doxorubicin, the treatment was discontinued due to the deterioration of her general condition. Finally, the patient entered a palliative care unit.

## 3. Discussion

Carcinosarcomas are highly aggressive neoplasms that can be mainly detected in the female genital tract [11]. Extragenital carcinosarcomas are extremely rare and mostly occur in the pelvic peritoneum [2], whereas various sites of the origin have been reported: cul-de-sac peritoneum, uterine serosa, colonic or rectal serosa, abdominal wall peritoneum, retroperitoneum, and omentum [12]. However, it is sometimes unclear as to where advanced peritoneal tumors emanate from within the abdominal cavity, as was the case in this patient.

Carcinosarcomas contain both malignant epithelial and sarcomatous elements. According to the histological characteristics of the sarcomatous element, carcinosarcomas are described as homologous or heterologous. Homologous carcinosarcomas have elements which are derived from Mullerian structures (e.g., endometrial stromal sarcoma, fibrosarcoma, or leiomyosarcoma). If the tumor has elements which are not normally found in Mullerian structures (e.g., cartilaginous, osseous, or rhabdomyoblastic), it is categorized as heterologous [13]. In the present case, the epithelial elements were similar to ovarian high-grade serous carcinoma, and the mesenchymal element resembled leiomyosarcoma. Therefore, this case of carcinosarcoma was diagnosed as a homologous type.

Primary peritoneal carcinoma is clinically and histologically similar to advanced epithelial ovarian carcinoma. The Gynecologic Oncology Group (GOG) has developed criteria to define primary peritoneal carcinoma: (1) both ovaries are normal in size or enlarged by a benign process; (2) the involvement in extraovarian sites is greater than the involvement on the surface of either ovary; (3) microscopically, the ovaries are not involved with the tumor or exhibit only serosal or cortical invasions with dimensions smaller than 5 × 5 mm; and (4) the histopathological and cytological characteristics of the tumor are predominantly of the serous type [14]. Although this case did not meet all criteria due to the lack of biopsy sampling from the ovary, we considered the most likely diagnosis to be primary peritoneal carcinosarcoma. In such advanced cases of peritoneal, ovarian, or fallopian cancer, laparoscopic surgery allows for the collection of tissue for definitive pathological diagnosis, observation of the uterus, ovaries and abdominal cavity, and the evaluation of the feasibility of complete cytoreductive surgery [15]. Moreover, patients can proceed immediately to neoadjuvant chemotherapy, compared with laparotomy.

Although there is only limited data regarding the management of primary peritoneal carcinosarsomas due to their rarity, surgical debulking is the main method of treatment. However, most cases have widespread metastasis at the time of presentation, and optimal tumor debulking is therefore difficult [16]. Based on recent reviews, platinum-based chemotherapy is the currently accepted systemic treatment, and the addition of paclitaxel or ifosfamide to platinum is recommended as a first-line treatment [11]. In regard to targeted therapy, Koyanagi et al. reported that chemotherapy including antivascular endothelial growth factor (VEGF) therapy with bevacizumab was markedly effective for carcinosarcomas arising from the Douglas' pouch [3]. In a study with ovarian carcinosarcoma patients, 4 of 9 (44%) tumors had expression of VEGF [13], yet the rate of BRCA1 and BRCA2 mutations in women with peritoneal or ovarian carcinosarcoma is unknown [11]. Because it is unclear as to whether a PARP inhibitor is effective on carcinosarcomas or not, we used olaparib in this case based on the patient's sensitivity to platinum. As a result, olaparib was effective, and she could continue olaparib therapy for 7 months without any decline in her quality of life (QOL). Although a dose reduction was needed due to anemia, no other significant adverse effects occurred.

Primary peritoneal carcinosarcomas are extremely rare tumors with a poor prognosis. In advanced cases, a laparoscopic biopsy is a useful method for the accurate diagnosis and observation of the abdominal cavity. Therefore, platinum-sensitive primary peritoneal carcinosarcoma patients should be considered for the further clinical development of olaparib, considering the effectiveness findings presented in this case.

## References

[1] Kanthan R., Senger J.-L. (2011). Uterine carcinosarcomas (malignant mixed Müllerian tumours): a review with special emphasis on the controversies in management. *Obstetrics and Gynecology International*.

[2] Shen D. H., Khoo U. S., Xue W. C. (2001). Primary peritoneal malignant mixed Müllerian tumors: a clinicopathologic, immunohistochemical, and genetic study. *Cancer*.

[3] Koyanagi T., To Y., Ando M. (2018). Primary peritoneal carcinosarcoma arising from the Douglas pouch: a case report. *Molecular and Clinical Oncology*.

[4] Shintaku M., Matsumoto T. (2001). Primary Mullerian carcinosarcoma of the retroperitoneum: report of a case. *International Journal of Gynecological Pathology*.

[5] Mikami M., Kuwabara Y., Tanaka K., Komiyama S., Ishikawa M., Hirose T. (2005). Malignant mixed Müllerian tumor of primary mesenteric origin. *International Journal of Gynecological Cancer*.

[6] Wang B., Ren K. W., Yang Y. C. (2016). Carcinosarcoma of the lesser omentum. *Medicine*.

[7] Komiyama S., Katabuchi H., Mikami M. (2016). Japan Society of Gynecologic Oncology guidelines 2015 for the treatment of ovarian cancer including primary peritoneal cancer and fallopian tube cancer. *International Journal of Clinical Oncology*.

[8] Ledermann J., Harter P., Gourley C. (2014). Olaparib maintenance therapy in patients with platinum-sensitive relapsed serous ovarian cancer: a preplanned retrospective analysis of outcomes by BRCA status in a randomised phase 2 trial. *The Lancet Oncology*.

[9] Sugarbaker P. H. (2019). Laparoscopy in the diagnosis and treatment of peritoneal metastases. *Annals of Laparoscopic and Endoscopic Surgery*.

[10] Eisenhauer E. A., Therasse P., Bogaerts J. (2009). New response evaluation criteria in solid tumours: revised RECIST guideline (version 1.1). *European Journal of Cancer*.

[11] Rauh-Hain J. A., Birrer M., del Carmen M. G. (2016). Carcinosarcoma of the ovary, fallopian tube, and peritoneum: Prognostic factors and treatment modalities. *Gynecologic Oncology*.

[12] Rajanbabu A., Ahmad S. Z., Vijaykumar D. K., Pavithran K., Kuriakose S. (2013). The significance of the site of origin in primary peritoneal carcinosarcoma: case report and literature review. *Ecancermedicalscience*.

[13] Zorzou M. P., Markaki S., Rodolakis A. (2005). Clinicopathological features of ovarian carcinosarcomas: a single institution experience. *Gynecologic Oncology*.

[14] Bloss J. D., Liao S. Y., Buller R. E. (1993). Extraovarian peritoneal serous papillary carcinoma: a case-control retrospective comparison to papillary adenocarcinoma of the ovary. *Gynecologic Oncology*.

[15] Hatae M., Onishi Y., Nakamura T., Wada T. (1998). The role of surgery for advanced epithelial ovarian cancer. *Cancer & Chemotherapy*.

[16] Hussein M. R., Hussein S. R. A., Abd-Elwahed A. R. (2009). Primary peritoneal malignant mixed mesodermal (Müllerian) tumor. *Tumori*.

